# Effect of thoracic paravertebral nerve block on the early postoperative rehabilitation in patients undergoing thoracoscopic radical lung cancer surgery

**DOI:** 10.1186/s12957-020-02071-8

**Published:** 2020-11-12

**Authors:** Kang Kang, Xing Meng, Bing Li, Jingli Yuan, Erhu Tian, Jiaqiang Zhang, Wei Zhang

**Affiliations:** 1 Department of Perioperative Medicine and Anesthesiology, Henan University People’s Hospital; Henan Provincial People’s Hospital, No. 7, Weiwu Road, Zhengzhou City, Henan Province China; 2 Department of Cardiology, Henan University People’s Hospital, No. 7, Weiwu Road, Zhengzhou City, Henan Province China

**Keywords:** Thoracic paravertebral nerve block, Lung cancer, Surgery, 6-min walking test

## Abstract

**Objective:**

To evaluate the effect of thoracic paravertebral nerve block on early postoperative rehabilitation in patients undergoing radical thoracoscopic surgery for lung cancer.

**Methods:**

Ninety patients scheduled for elective video-assisted thoracoscopic lobectomy of lung cancer were divided into 2 groups: the general anesthesia group (GA group, *n* = 45) and the TPVB group (TP group, *n* = 45). The primary outcome was the decline rate of the 6-min walking test (6MWT); the second outcomes were as follows: absolute value and the completion rate of 6MWT, postoperative analgesia deficiency and pain scores, oxycodone consumption, sleep quality, the incidence of postoperative pulmonary complications, and the hospital stay.

**Results:**

Compared with the GA group, the TP group had a lower decline rate of the 6MWT on POD1 and POD2. The walking distance on POD1 and POD2 in the TP group was significantly longer than that in the GA group; the completion rate at POD1 in the TP group was higher than that in the GA group. The pain scores and oxycodone consumption at POD1 in the TP group were lower than the GA group. The sleep quality in the TP group was higher than the GA group.

**Conclusions:**

TPVB can significantly improve postoperative rehabilitation in patients undergoing thoracoscopic radical lung cancer surgery, which is helpful for promoting the early recovery of patients.

**Trial registration:**

Chinese Clinical Trial Registry, ChiCTR1900026213. Registered 26 Sept. 2019, http://www.chictr.org.cn/showproj.aspx?proj=43733.

## Introduction

Lung cancer has a high incidence and accounts for a large proportion of thoracic surgeries. Radical resection of lung cancer is traumatic, and early postoperative pain is obvious, which is not conducive to rapid recovery [1, 2]. The improvement of anesthesia factors has positive significance for promoting the rehabilitation of patients. Thoracic paravertebral nerve block (TPVB) is an important method of multimodal analgesia that can reduce acute pain and opioid dosage after thoracoscopic surgery [3–5]. Early postoperative bedside activities have been recognized as important to recovery after surgery, which can be hampered by severe pain. Reduced pain can help patients carry out postoperative rehabilitation training.

The 6-min walking test (6MWT) is a simple and effective method for evaluating patients’ cardiopulmonary function, which has a guiding significance for the quality of postoperative recovery from lung cancer [6–11]. The effect of TPVB on the quality of early postoperative rehabilitation in patients undergoing radical surgery for lung cancer remains unclear.

In this study, patients who underwent thoracoscopic radical lung cancer surgery (thoracoscopic lobectomy plus systemic lymph node dissection) [1] were enrolled. The 6MWT and other measurements were selected to evaluate the effect of TPVB on the early rehabilitation quality of patients undergoing radical resection for lung cancer. It was hypothesized that early postoperative rehabilitation could be enhanced by TPVB.

## Materials and methods

### Study design and setting

This randomized, double-blind study enrolled 90 patients from Oct. 2019 to May 2020. This prospective study was approved by the Ethics Committee of Henan Provincial People’s Hospital [Ethics approval number: 2019-Lunshen-41]. Written informed consent and information release approvals were obtained from all patients prior to their participation in the study. Trial registration—Chinese Clinical Trial Registry, ChiCTR1900026213. Registered 26 Sept. 2019-Prospectively registered, http://www.chictr.org.cn/showproj.aspx?proj=43733. The study protocol compline with the 1975 Declaration of Helsinki.

### Characteristics of participants

Ninety patients with lung cancer, aged 18-65 years, with a BMI of 18-25 kg/m^2^ and an ASA status of I-III, were selected. The patients were randomly divided into two groups: the general anesthesia group (GA group, *n* = 45) and the TPVB combined with general anesthesia group (TP group, *n* = 45).

#### Inclusion criteria

Confirmed preoperative diagnosis; no history of diabetes, blood disease, or other metabolic disorders; no chronic obstructive or (and) restrictive lung disease; a forced vital capacity > 80% of the predicted value and a first second expiratory rate > 70% of the predicted value; and no smoking history within 2 weeks before the operation.

#### Exclusion criteria

Communication difficulties; inability to cooperate with researchers; history of preoperative radiotherapy and chemotherapy; severe cardio-cerebrovascular disease; previous history of other operations; refusal to participate in the trial; drop-out from the trial; and data loss.

### Preoperative preparations and anesthesia protocol

No patients received pretreatment before admission to the operating room. All patients were monitored by electrocardiography, pulse oximetry, invasive blood pressure recordings, and bispectral index values.

### Operation of TPVB

TPVB was administered by using S-Nerve ultrasound (SonoSite, USA). Two points (T4-5 and T6-7) were selected for blocking, with 10 ml of 0.5% ropivacaine at each point. The success of the block was verified by using skin temperature sensation and tactile sensation in the corresponding area with alcohol-soaked cotton balls [12].

The blinding principle was well controlled in the present study. First, patients were delivered to the pre-anesthesia room. An anesthesiologist who was blinded to this study prepared the drugs and TPVB. All the catheter insertion sites were covered with a film dressing to conceal which approach was being used.

### Anesthesia induction

After intravenous injection of 0.08-0.12 mg/kg midazolam, 0.1-1.0 g/kg sufentanil, 0.2-0.6 mg/kg etomidate, and 0.6-0.9 mg/kg rocuronium, a double-lumen bronchial tube was inserted. Mechanical ventilation was performed after induction: FiO_2_ 70%, oxygen flow 1.0-1.5 L/min, V_T_ 6-8 ml/kg, RR 10-14 times/min, and I:E 1:2. During single-lung ventilation, the RR was 12-16 times/min, the other parameters remained unchanged, P_ET_CO_2_ was maintained at 35-45 mmHg, and the peak airway pressure was maintained lower than 30 cm H_2_O.

### Anesthesia maintenance

Both groups received intravenous infusion of propofol and remifentanil to maintain the depth of anesthesia and intermittent intravenous infusion of cis-atracurium to maintain muscle relaxation. The infusion rate of propofol was adjusted to maintain a BIS of 40-50, and the fluctuation ranges of the HR and MAP were maintained at no more than 20% of the baseline values. The intraoperative intravenous infusion of Ringer’s solution of sodium lactate was 1-3 ml/kg/h. The postoperative analgesia pump was consistent between the two groups. In addition, 1 mg/kg oxycodone was added to postoperative patient-controlled analgesia pump for postoperative analgesia.

### Measurements

A respiratory therapist who was unaware of the study performed a 6MWT for each patient on the day before surgery (POD0), the first day after the operation (POD1), the second day after the operation (POD2), and the third day after the operation (POD3) [9–11].

The primary outcome of this study was the decline rate of the 6MWT on POD1 [(6MWT_POD0_−6MWT_POD1_)/6MWT_POD0_].

The secondary outcomes of this study were as follows: the absolute value of walking distance; the completion rate of the test (completion rate = number of completed cases per group/total number of cases per group), with the following reasons why patients could not complete the 6MWT: (1) chest pain, (2) intolerable dyspnea, (3) leg cramps, (4) staggering, (5) diaphoresis, and (6) a pale or ashen appearance [13]; the insufficiency of postoperative analgesia within 24 h (VAS ≥ 4 at rest); pain scores at POD1, POD2, and POD3; oxycodone consumption in the first 24 h after surgery; sleep quality (Pittsburgh Sleep Quality Index (PSQI) scale was used to evaluate sleep quality) [14, 15]; the incidence of postoperative pulmonary complications [16, 17]; and the hospital stay. According to the study by Sun et al. [12], the complications directly related to TPVB were also observed.

### Sample size calculation

The sample size was calculated with “Power and Sample Size.com,” an online power and sample size calculator. The primary objective of this study was the effect of TPVB on the decline rate of the 6MWT on POD1. According to our preliminary results, the decline rate of the 6MWT on POD1 in the GA group was 66%. We hypothesized that the decline rate would have a 50% improvement in the TP group. A sample size of 33 patients in each arm was calculated by using a power of 0.8, a *P* value of 0.05, and a type-I error of 5%. Taking into account patients lost to follow-up, we enrolled 45 patients in each group.

### Statistical analysis

SPSS 21.0 statistical software was used for data analysis. The measurement data for a normal distribution were expressed as the mean ± standard deviation; the non-normal data were expressed as the median and interquartile range; and the normal count data were compared with the chi-square test, the non-normal data were compared with the rank-sum test, and the repeated data were compared with repeated measures ANOVA. *p* < 0.05 was regarded as statistically significant.

## Results

### Characteristics of the patients

**Fig. 1:**
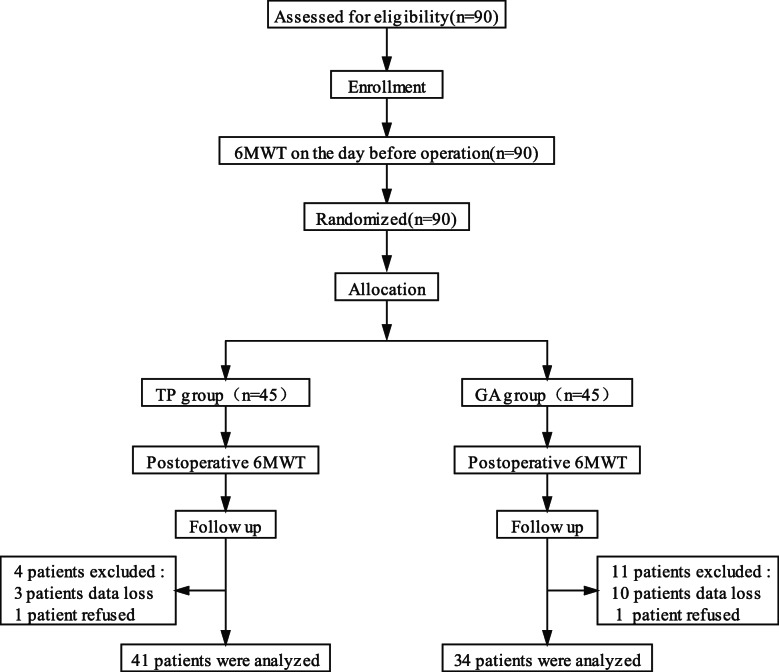
Study flow diagram

In this study, 90 patients were included. Due to changes in surgical methods, intraoperative blood transfusion, and failure to implement the 6MWT (non-patient factors), 15 patients were excluded. Finally, 75 patients were analyzed, 41 in the TP group and 34 in the GA group, as shown in Fig. 1. There was no significant difference in the general conditions between the two groups (*p* > 0.05), as shown in Table 1. No complications directly related to TPVB occurred.

**Table 1:** Characteristics of the patients

	TP group (*n* = 41)	GA group (*n* = 34)
Age (year)	51.6 ± 11.0	56.1 ± 9.8
Males/females	21/20	16/18
BMI (kg/m^2^)	23.7 ± 3.2	24.0 ± 2.2
ASA (I/II/III)	1/39/1	0/34/0
Surgery duration (min)	204 ± 61	179 ± 60
Estimated blood loss (ml)	100 (100, 100)	100 (50, 100)
Urinary output (ml)	300 (200, 500)	325 (100, 500)
Fluid input (ml)	1300 (1000, 1500)	1500 (1000, 1700)

### Results of the 6MWT in the two groups

**Fig. 2:**
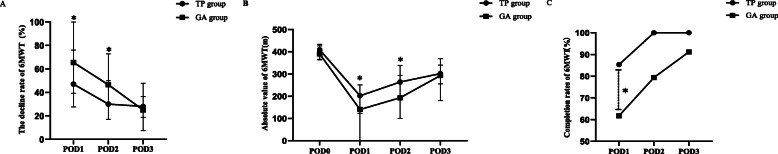
Results of the 6MWT in the two groups. Comparison of 6MWT between the two groups. **a** The decline rate of 6MWT in the two groups at different time points. Compared with GA group, TP group had a lower decline rate of 6MWT on the POD1 and POD2. **b** The absolute value of 6MWT at different time points between the two groups. The 6MWT distance on the POD1 and POD2 was significantly greater in the TP group than that GA group. **c** The completion rate of 6MWT between the two groups. The completion rate on POD1 was higher in the TP group than the GA group. *p < 0.05, compared with the GA group

There was no significant difference in the 6MWT on POD0 between the two groups (*p* > 0.05). Compared with the GA group, the TP group had a lower decline rate of the 6MWT on POD1 and POD2 (*p* < 0.05), as shown in Fig. 2a. The 6MWT distance on POD1 and POD2 in the TP group was significantly longer than that in the GA group (*p* < 0.05), as shown in Fig. 2b. The completion rate on POD1 in the TP group was higher than that in the GA group (*p* < 0.05), as shown in Fig. 2c.

### Postoperative complications, analgesia, sleep quality, and hospital stay

Postoperative pulmonary complications (number and rate) occurred in 7 patients (17.0%) and 8 patients (23.5%) in the TP group and GA group, respectively. There was no statistically significant difference between the two groups. The incidence (number and rate) of postoperative analgesia deficiency in the two groups was as follows: TP group (2 cases, 4.88%) and GA group (2 cases, 5.88%). There was no significant difference in the incidence of postoperative analgesia deficiency between the two groups (*p* > 0.05). There was no significant difference in hospital stay between the TP group and GA group (*p* > 0.05), as shown in Table 2.

**Table 2:** Comparison of postoperative pulmonary complications, analgesia and length of hospital stay

Group	Pulmonary complication (number and rate)	Insufficient analgesia (number and rate)	Hospitalization time (day)
TP	7 (17.07%)	2 (4.88%)	6.4 ± 1.8
GA	8 (23.53%)	2 (5.88%)	6.0 ± 1.5

The pain scores at POD1 in the TP group were lower than the GA group (*p* < 0.05). The PSQI score in the TP group was higher than the GA group (*p* < 0.05). Patients in the TP group received less oxycodone than the GA group in the first 24 h after surgery (*p* < 0.05), as shown in Table 3.

**Table 3:** Comparison of VAS score, PSQI and oxycodone consumption between groups

	Group	POD0	POD1	POD2	POD3
VAS score	TP		2 (1, 2)*	1 (1, 2)	1 (0, 2)
	GA		3 (2, 3)	2 (2, 3)	2 (1, 2)
PSQI	TP	2 (2, 3)	5 (4, 5)*	5 (4, 5)*	4 (4, 5)*
	GA	3 (2, 3)	8 (7, 8)	7 (6, 7)	6 (6, 7)
Consumption of oxycodone (mg)	TP		31.2 (30, 32)*		
	GA		40.0 (37.2, 40.8)		

Data are presented as median (interquartile range)

*POD0* the day before surgery; *POD1* the first day after the operation; *POD2* the second day after the operation; *POD3* the third day after the operation; *PSQI* Pittsburgh Sleep Quality Index

*p < 0.05, compared with the GA group

## Discussion

The main finding of this study is that the decline rate of the 6MWT on POD1 could be improved by TPVB after radical thoracoscopic surgery for lung cancer. To the best of our knowledge, this is the first study to discuss the effect of TPVB on the 6MWT. The absolute walking distance and completion rate benefited from TPVB. Based on the conclusions, early rehabilitation after thoracoscopic surgery can be enhanced by TPVB.

In this study, the same group of surgeons was selected to balance the effects of different surgical skills and perioperative management. The same professional respiratory therapist who was not aware of the study was selected for performing the 6MWT to ensure the authenticity and objectivity of the results. To ensure the objectivity of the data, anesthesiologists and professional anesthesiology nurses who were not aware of the experimental groups were selected for postoperative follow-up and statistical analysis of the data.

Early bedside activities after surgery are helpful for rapid recovery and can provide the basic conditions for postoperative recovery. The 6MWT is a simple and effective method to evaluate patients’ cardiopulmonary function, which can reflect exercise endurance and predict the occurrence of postoperative pulmonary complications to a certain extent [7, 13]. It is one of the bases for formulating a postoperative rehabilitation plan and has a guiding significance for the postoperative rehabilitation quality of lung cancer patients.

Three main aspects of 6MWT were evaluated in this study: decline rate of walking distance, absolute walking distance, and completion rate. The results indicated that the decline rate of the 6MWT on POD1 and POD2 was lower in the TP group than in the GA group, in addition to that, the absolute distance of the 6MWT in the TP group was significantly greater than that in the GA group on POD1 and POD2, the completion rate of the 6MWT on POD1 was higher in the TP group than in the GA group. The decline rate of the walking test, the absolute value, and the completion rate were consistent, especially on POD1. These results indicate that thoracic paravertebral nerve block can improve the early mobility in patients undergoing radical resection for lung cancer and can provide the necessary conditions for promoting early recovery.

Pulmonary complications represent an important group of complications after radical resection for lung cancer and can prolong the hospital stay. One retrospective study showed that general anesthesia combined with TPVB may reduce postoperative pulmonary complications [18], and other studies have shown that TPVB can shorten the postoperative hospital stay [19, 20]. Our study showed that there was no difference in postoperative pulmonary complications or hospital stay between the two groups. The following may be reasons for these results: (1) there are many influencing factors for pulmonary complications, which require the comprehensive application of multiple strategies; (2) there are differences in the methods of TPVB.

As is well known, pneumothorax, puncturing of blood vessels, and local hematoma are common complications of TPVB. According to the study by Sun et al. [12], the complications directly related to TPVB were also observed. No complications directly related to TPVB occurred in our study.

Numerous studies have shown that TPVB can reduce postoperative acute pain [21, 22]. Our results showed that the pain scores at POD1 in the TP group were lower than the GA group. The oxycodone consumption in the TP group was less than the GA group. TPVB alleviated the acute pain at POD1 and decreased the opioid consumption. However, the occurrence of postoperative insufficient analgesia was evaluated in this study. There was no difference in the occurrence of postoperative insufficient analgesia between the two groups. The possible reasons were speculated as follows: (1) the incidence of postoperative overall insufficient analgesia tends to decrease after minimally invasive surgery; (2) the analgesic effect was evaluated 24 h after the operation, and the earlier pain level within 24 h after the operation was not evaluated; and (3) the index of analgesia evaluation in this study was resting pain, and no evaluation was made for motion pain. Although there was no difference in the incidence of postoperative analgesia deficiency between the two groups on POD1, patients in the TP group, who had a better performance on the 6MWT, benefited from TPVB.

High quality of sleep helps patients recover after surgery. However, the sleep quality after surgery is usually poor. Postoperative pain is one of the important causes of poor postoperative sleep quality [23]. Our results showed that the sleep quality was improved due to the TPVB. We speculate that TPVB’s relief of postoperative pain may be one of the reasons for its improvement of postoperative sleep quality.

At the same time, we also found that the differences between the two groups were concentrated on POD1 and POD2 after the operation. Although the results showed that TPVB could not significantly improve pulmonary complications or hospital stay, TPVB could promote the early rehabilitation of patients and supply a foundation for rapid rehabilitation.

There are several limitations in this study. Firstly, the sample size was relatively small due to the single-center study. Multicenter studies should be conducted in the future. Secondly, postoperative recovery includes multiple influencing factors. Only TPVB was discussed in this study. Multimode rehabilitation strategies should be administered to achieve the purpose of enhanced recovery. Thirdly, our results are only valid for thoracoscopic radical lung cancer surgery, and not for all thoracic surgeries.

Overall, the 6MWT could be improved by TPVB in this study. TPVB can significantly improve postoperative rehabilitation in patients undergoing thoracoscopic radical lung cancer surgery, which is helpful for promoting the early recovery of patients.

## Data Availability

The datasets used and analyzed during the current study are available from the corresponding author on reasonable request.

## Abbreviations

ASA: American Society of Anesthesiologists
BMI: Body mass index
FiO_2_: Fraction of inspiration oxygen
VT: Tidal volume
RR: Respiratory rate
I:E: Inspiratory:expiratory
BIS: Bispectral index
P_ET_CO_2_: End-tidal carbon dioxide partial pressure
HR: Heart rate
MAP: Mean arterial pressure
6MWT: 6-min walking test
VATS: Video-assisted thoracoscopic surgery
POD0: The day before surgery
POD1: The first day after the operation
POD2: The second day after the operation
POD3: The third day after the operation
